# Preparation and Properties of Dynamic Covalent-Based Epoxidized Soybean Oil-Derived UV-Curable Resin

**DOI:** 10.3390/polym18172055

**Published:** 2026-08-24

**Authors:** Wei Wang, Wen Lei, Han Luo, Wangwang Yu, Yong Chen

**Affiliations:** 1 College of Science, Nanjing Forestry University, Nanjing 210037, China; 2 School of Mechanical Engineering, Nanjing University of Industry Technology, Nanjing 210023, China

**Keywords:** UV curable resin, epoxidized soybean oil, dynamic covalent bond, self-healing

## Abstract

To develop ultraviolet (UV)-curable resin with excellent mechanical, thermal-resistant and self-healing properties, epoxidized soybean oil was utilized as a bio-based raw material in this paper, and its epoxy groups were ring-opened and modified with methanol and tert-butyl acetoacetate to introduce hydroxyl groups and flexible segments, yielding a functionalized polyol, which was reacted with isophorone diisocyanate to prepare a hydroxyl-terminated polyurethane prepolymer containing dynamic covalent bonds. The prepolymer was further end-capped with hydroxyethyl acrylate to obtain a UV-curable polyurethane acrylate resin. The physico-mechanical properties and self-healing efficiency of the samples were systematically investigated. The results showed that the prepared specimens had efficient self-healing capability and could achieve efficient repair of damaged interfaces through appropriate heat treatment without the need for external catalysts; the onset decomposition temperatures of all the samples were greater than 225 °C, demonstrating good thermal stability; the tensile strength, tensile modulus, flexural strength and flexural modulus could be as great as 31.1 MPa, 393.7 MPa, 29.6 MPa and 851.6 MPa, respectively. All these indicated that the prepared samples had good overall performances. This study provides a new strategy for the design and preparation of self-healing photocurable resins based on renewable resources.

## 1. Introduction

Ultraviolet (UV) curing technology is widely applied in coatings, inks, adhesives, and other areas due to its advantages of rapid curing at ambient temperature, less energy use, and low VOC emissions [1,2]. However, microcracks generated in traditional UV-cured materials during service are difficult to be repaired, leading to a decline in mechanical performance and service life [3,4]. Inspired by the self-healing phenomena observed in living organisms, endowing materials with intrinsic self-healing capabilities has emerged as a cutting-edge strategy for extending material lifespan, enhancing reliability, and improving safety [5,6]. Incorporation of dynamic covalent bonds (such as Diels–Alder bonds, disulfide bonds, dynamic ester bonds, etc.) into polymer networks is suitable for preparing self-healing materials with better mechanical properties and is thus attracting more and more attention [7,8,9,10].

With the deepening implementation of sustainable development strategies, the development of high-performance, multifunctional, and environmentally friendly polymer materials has become a crucial direction in the fields of chemistry and materials science [11,12]. Substitution of traditional petroleum-based raw materials with abundant and renewable resources is an inevitable trend in green chemistry [13,14]. As a typical example, epoxidized soybean oil (ESO), a low-cost triglyceride with epoxidized fatty acid chains, is often converted into polyols via ring-opening reactions for the synthesis of polymers such as polyurethanes [15,16]. However, conventional ring-opening products (e.g., alcoholysis products) often exhibit limited functionality and are not readily suitable for introducing dynamic properties. For these, extensive research has been conducted on the alcoholysis modification of ESO, and preparation of its UV-curable resins with self-healing ability based on dynamic covalent bonds, but most existing systems suffer from the following limitations: (1) Limited functionality: ESO modifications typically only introduce hydroxyl groups, lacking deliberate design for dynamic properties. (2) Complex processes: constructing self-healing networks often requires multi-step synthesis or the addition of exogenous catalysts. (3) Difficulty in balancing properties: the rigidity of bio-based raw materials may increase brittleness, while efficient self-healing often requires good segmental mobility [17,18]. Therefore, how to synergistically integrate bio-based raw materials, dynamic self-healing networks, and efficient UV-curing technology through concise molecular design, while maintaining good mechanical properties and endowing the material with efficient self-healing capability, remains a significant challenge [19].

Based on this, an innovative strategy of “one-step ring-opening modification followed by two-step network construction” was proposed in this study, aiming to prepare a high-performance self-healing UV-curable material. First, using ESO as the bio-based core, synergistic ring-opening modification was carried out with methanol and tert-butyl acetoacetate (t-BAA) to synthesize a functionalized polyol (ESO-tBAA) in a one-pot process, which contained both reactive hydroxyl groups and potential dynamic ester bond precursors (acetoacetate groups). Subsequently, this polyol was combined with flexible polyether polytetrahydrofuran (PTMG) as a mixed soft segment [20,21] and reacted with isophorone diisocyanate (IPDI) to construct a polyurethane prepolymer (EOtB_x_P) containing dynamic covalent bonds. Finally, hydroxyethyl acrylate (HEA) was introduced via end-capping to incorporate photocurable groups [22], yielding a UV-curable polyurethane acrylate resin. The effects of ring-opening conditions and soft-segment ratios on the network structure, mechanical properties, thermal stability, and self-healing behavior of the material were systematically investigated. The structure–property relationships were elucidated in depth, providing a new design concept and practical basis for developing a new generation of green, smart, and repairable advanced coating materials.

Compared with previously reported UV-curable systems based on ESO, our work addresses several key limitations. For instance, many existing ESO-based self-healing materials either rely on commercially available polyols without deliberate design for dynamic properties [23,24,25,26] or require complex multi-step synthesis and external catalysts to trigger the healing process [27,28,29,30]. In contrast, our proposed “one-step ring-opening + two-step network construction” strategy offers distinct advantages: (i) Efficiency: The one-pot ring-opening modification of ESO with methanol and t-BAA efficiently introduces both hydroxyl groups and dynamic ester precursors, simplifying the synthesis. (ii) Catalyst-free Self-Healing: The resulting crosslinked network, containing dynamic amide bonds from the reaction of acetoacetate with isocyanate, undergoes efficient transamidation reactions upon heating without the need for any external catalyst, which is a significant improvement over many catalyst-dependent vitrimer systems. (iii) Property Tuning: The strategy allows for facile tuning of the network structure by simply adjusting the ratio of dynamic components (ESO-tBAA) and PTMG, enabling an optimal balance between mechanical strength and self-healing capability. This integrated molecular design provides a novel and practical pathway for developing advanced, sustainable polymer materials.

## 2. Materials and Methods

### 2.1. Materials

Epoxidized soybean oil (ESO, 98.9%) was supplied by Jiangsu Donghu Bioenergy Plant Co., Ltd., Nanjing, China. Isophorone diisocyanate (IPDI, 96%), polytetrahydrofuran (PTMG, 99.5%), hydroxyethyl acrylate (HEA, 99.5%), methanol (99%), and dibutyltin dilaurate (DBTDL, 99%) were purchased from Macklin (Shanghai) Chemical Reagent Co., Ltd., Shanghai, China; 2-Hydroxy-2-methylpropiophenone (D1173, 99%) was supplied by Tianjin Chemical Reagent Co., Ltd. Tianjin, China; Tert-butyl acetoacetate (t-BAA, 99%) and the other chemical reagents were obtained from Nanjing Wanqing Chemical & Glass Co., Ltd., Nanjing, China; All chemicals were used as received without further purification.

### 2.2. Preparation of the Functionalized Polyol (ESO-tBAA)

Epoxidized soybean oil (ESO, 55 g) and methanol (85 g) were mixed in a 250 mL round-bottom flask, and methanesulfonic acid (0.1 wt% relative to ESO) was added as a catalyst. The mixture was heated at 65 °C with magnetic stirring for 2 h, then quenched to room temperature. The product was extracted with ethyl acetate and washed three times with saturated sodium chloride solution. After removal of the organic solvent by rotary evaporation, a colorless oil, designated ESO-MeOH, was obtained. This intermediate was then dissolved together with tert-butyl acetoacetate (30 g) in a 250 mL flask equipped with a reflux condenser. Under a nitrogen atmosphere, the reaction was heated to 110 °C with magnetic stirring and maintained for 3 h. Subsequently, the released tert-butanol was removed under reduced pressure at 100–110 °C to afford a yellow oily product, ESO-tBAA, with a yield of 85%.

### 2.3. Polymerization and UV Curing of Polyurethane Acrylate (EOtB_x_P)

**Figure 1:**
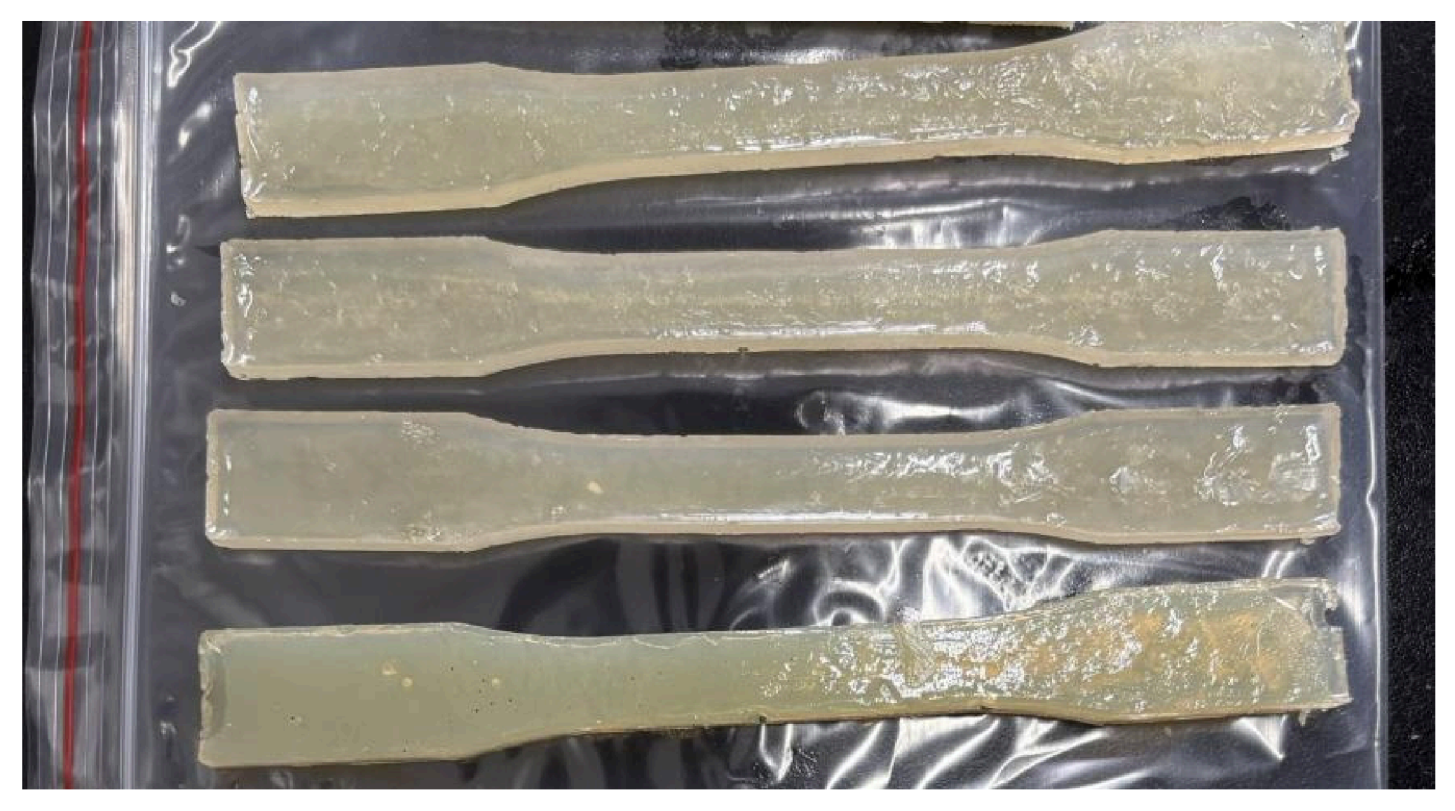
Photos of UV-cured EOtB_x_P samples.

**Figure 2:**
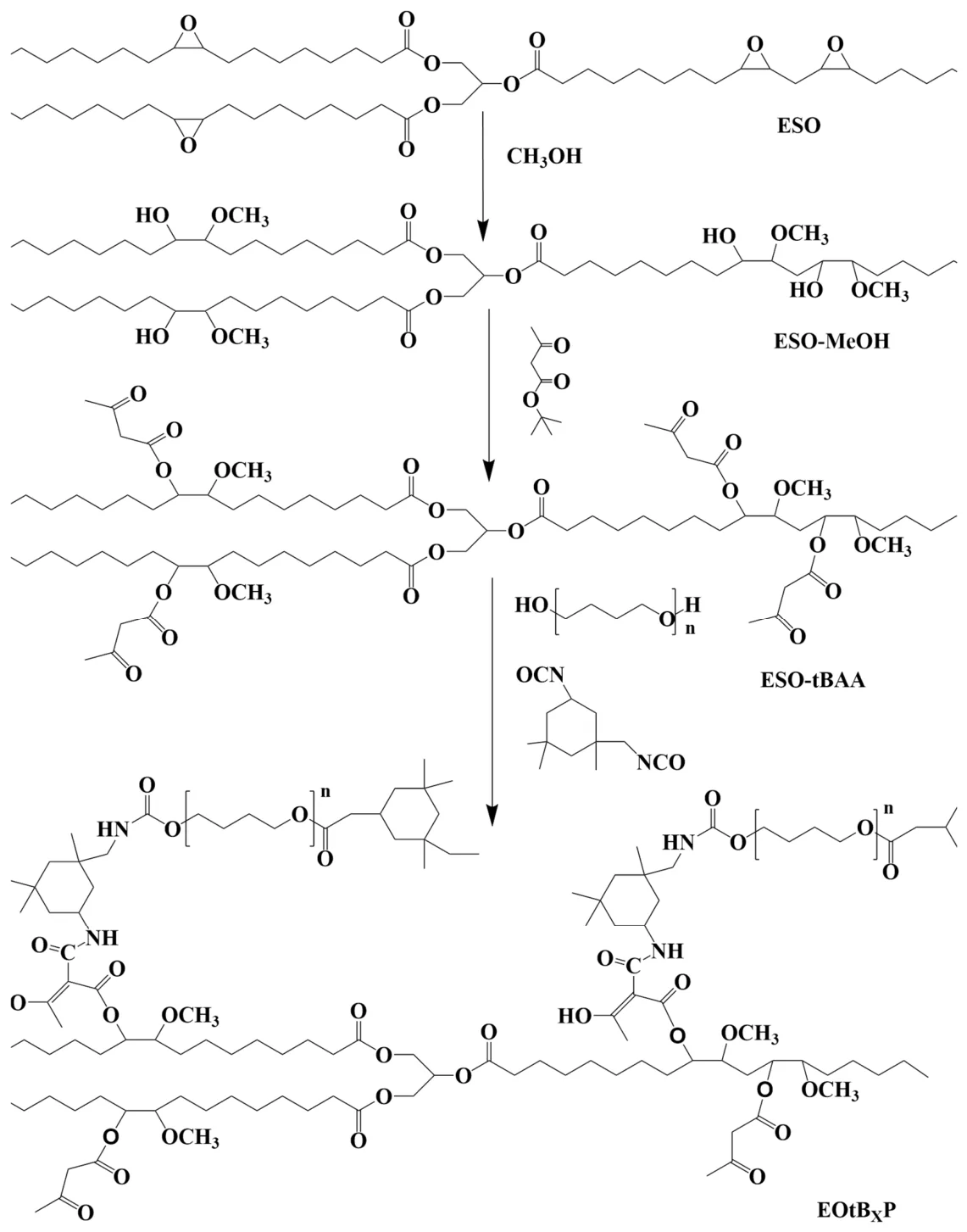
Synthesis route of EOtB_x_P.

The photocurable resin was prepared according to the formulations outlined in Table 1. Into a three-neck flask were added 8.5 g of polytetrahydrofuran (PTMG), 20.5 g of isophorone diisocyanate (IPDI), 0.02 g of the catalyst dibutyltin dilaurate (DBTDL), and 30 g of the functionalized polyol ESO-tBAA to obtain a polyurethane prepolymer. The mixture was mechanically stirred at 55 °C for 3 h. After completion of the reaction, excess solvent was removed under reduced pressure using a rotary evaporator, yielding a light yellow viscous liquid product, designated as EOtB_x_P. To the obtained photosensitive prepolymer, hydroxyethyl acrylate (HEA) was added as a reactive diluent, followed by the addition of 3 wt.% of the photoinitiator 2-hydroxy-2-methylpropiophenone(D1173). The mixture was stirred at room temperature for 30 min and then was exposed to ultraviolet light with a wavelength of 395 nm and an irradiance of 100 mW/cm^2^ for a duration of 10 min to obtain the UV-cured sample, as demonstrated in Figure 1. The experimental principle is illustrated in Figure 2.

### 2.4. Characterization

#### 2.4.1. FT-IR and NMR Analyses

Infrared spectra were obtained using a Nicolet iS10 spectrometer (Thermo Scientific, Waltham, MA, USA) in attenuated total reflectance (ATR) mode; all measurements were performed over the spectral range of 500–4000 cm^−1^ with a resolution of 4 cm^−1^. For each sample, 32 scans were co-added to improve the signal-to-noise ratio. The sample surface was pressed against the ATR crystal, and the collected spectra were baseline corrected for further analysis.

^1^H NMR of the chemicals was recorded on a Bruker AV400 spectrometer (Bruker, Rheinstetten, Germany) with Chloroform-d as the solvent. The conversion of epoxy groups was calculated from the ^1^H NMR spectra by comparing the integrated peak areas of the oxirane protons (δ = 2.8–3.0 ppm) relative to the internal reference peak of the glycerol backbone methine protons (δ = 5.0–5.4 ppm). The conversion was determined according to the following Equation (1):

(1) $$\mathrm{Conversion}\;(\%)=\left(1-\frac{A_{2.8–3.0}/A_{5.0–5.4}(Pre\text{-}reaction)}{A_{2.8–3.0}/A_{5.0–5.4}(Post\text{-}reaction)}\right)\times 100\%$$

#### 2.4.2. Gel Content Measurement

The gel content of the cured samples was determined by Soxhlet extraction. The cured specimen was first dried in an oven at 60 °C for 24 h and accurately weighed (denoted as m_0_). It was then extracted with acetone in a Soxhlet apparatus for 24 h. After extraction, the sample was dried again in a vacuum oven at 60 °C for 24 h and re-weighed (denoted as m_1_). The gel content (C_gel_) was calculated according to Equation (2):

(2) $$C_{\mathrm{gel}=}\frac{m_{1}}{m_{0}}\times 100\%$$

For each formulation, three independent samples were tested, and the average values were reported.

#### 2.4.3. Thermal Stability Assessment

Thermal stability was evaluated using a TG 209 F1 thermogravimetric analyzer (Netzsch, Selb, Germany). Approximately 6–8 mg of each sample was heated from 20 °C to 550 °C under a nitrogen atmosphere at a heating rate of 20 °C/min with a gas flow rate of 20 mL/min. The onset decomposition temperature (T_5%_) and the temperature at the maximum rate of mass loss (T_p_) were recorded to assess the thermal stability of the UV-cured samples.

#### 2.4.4. Mechanical Property Determination

Tensile and flexural tests were conducted on an E44.304 universal testing machine (MTS Industrial Systems, Shanghai, China) with a 20 kN load cell at ambient temperature. Tensile properties were tested according to ASTM D638 [31] at a crosshead speed of 10mm/min. Flexural properties were tested according to ASTM D790 [32] at a crosshead speed of 5 mm/min. For each formulation, a minimum of five specimens were tested to ensure statistical significance, and the average values along with standard deviations are reported in Table 4.

#### 2.4.5. Self-Healing Performance Measurement

The self-healing capability of the UV-cured samples was evaluated by monitoring the recovery of artificial scratches. A thin blade was used to create scratches on the cured film (coated on a glass slide, thickness ≈ 200 μm), and the initial scratch width (D_1_) was measured using an optical microscope. The scratched sample was then heated at 180 °C for a defined period, after which the scratch width (D_2_) was measured again. The healing efficiency (R_r_) was calculated using Equation (3):

(3) $$R_{r}=\frac{D_{1}-D_{2}}{D_{1}}\times 100\%$$

#### 2.4.6. Shape Memory Performance Observation

The shape memory performance was evaluated on rectangular specimens by five steps. Step 1, the sample was heated at 120 °C for 10 min, quickly twisted into an “S” shape, and then immediately immersed in cold water at 25 °C to observe whether the shape could be fixed. Step 2, the twisted sample was reheated to 120 °C to observe whether its original shape could be recovered. Step 3, the sample was wrapped into a “W” shape, heated at 150 °C for 10 min, and rapidly cooled in cold water at 25 °C to observe whether the “W” shape could be locked. Step 4, the sample was reheated to 120 °C under external force to observe whether the “W” shaped sample could be returned to its rectangular shape. Step 5, the external force was removed to observe whether the sample could be quickly recovered to the “W” shape at 150 °C.

#### 2.4.7. Wettability Measurement

The wettability of distilled water on the surface of each sample was characterized by measuring the water contact angles at room temperature using a DSA100 contact angle goniometer (KRÜSS, Borsteler Chaussee, Hamburg, Germany). A 5 μL droplet of distilled water was placed on the cured sample’s surface, and the contact angle θ was recorded after 15 s. Measurements were taken at multiple locations on each sample.

## 3. Results and Discussion

### 3.1. Spectroscopic Evidences of EOtB_x_P

**Figure 3:**
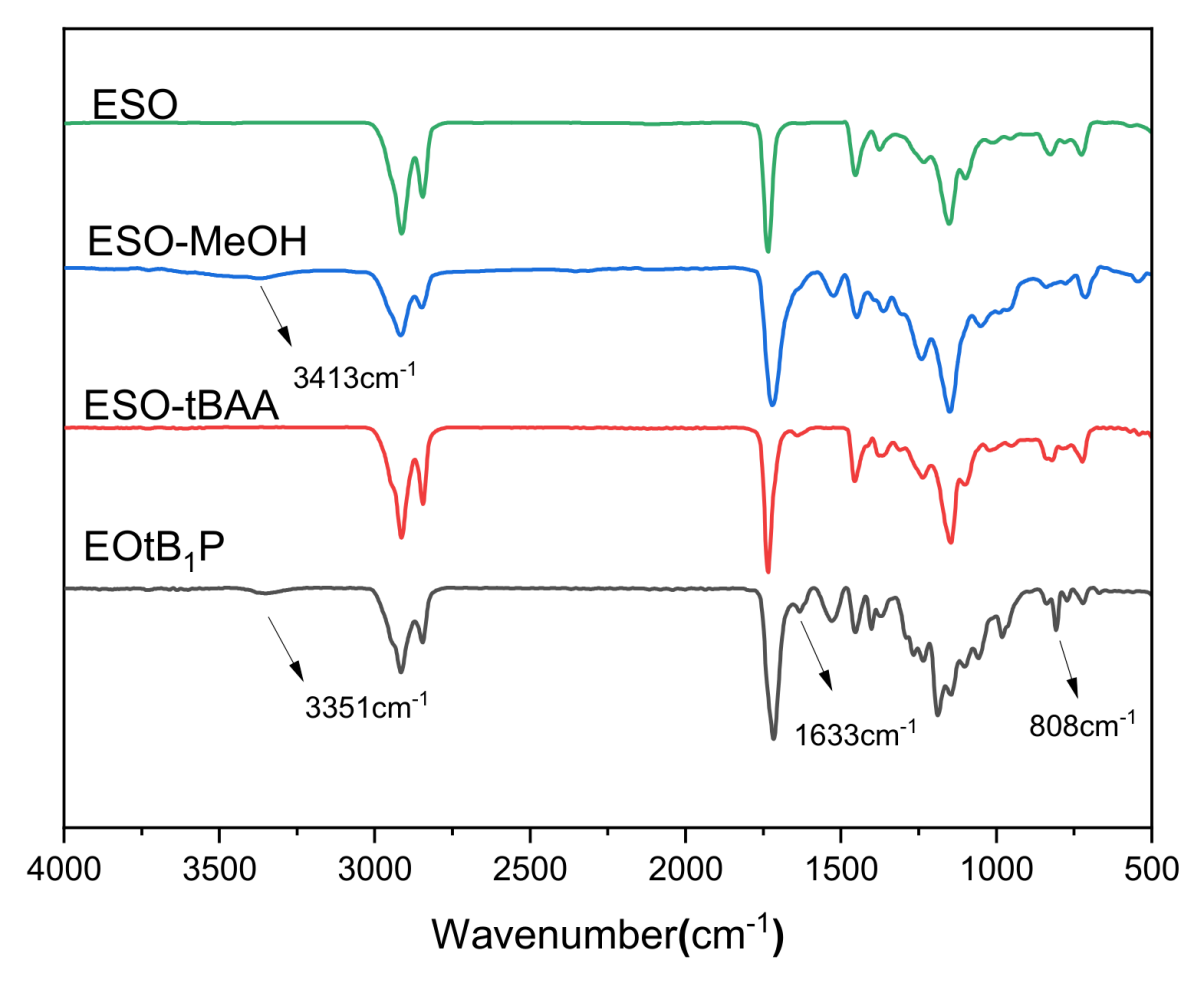
FTIR spectra of ESO, ESO-MeOH, ESO-tBAA, and EOtB_x_P.

In the FTIR spectrum of EOtB_x_P (Figure 3), the successful synthesis of the urethane acrylate prepolymer was confirmed by several key spectral features. The appearance of a new, sharp absorption band at 3351 cm^−1^, assigned to the N-H stretching vibration of the urethane linkage, along with the band at 1535 cm^−1^ (N-H bending coupled with C-N stretching), indicated the formation of the urethane group from the reaction between the hydroxyl groups of the polyols and the isocyanate groups of IPDI [33,34]. Crucially, the complete disappearance of the characteristic absorption band at 2270 cm^−1^ (asymmetric stretching of the free isocyanate, -NCO) served as definitive evidence that the isocyanate groups had been fully consumed during the prepolymerization step, ensuring a stoichiometrically balanced system prior to UV curing [35,36]. Furthermore, the presence of the C=C bending vibration at 808 cm^−1^ confirmed the successful end-capping of the prepolymer with HEA, thereby introducing the necessary photoreactive functional groups. The shift and intensity changes of the C=O stretching band at 1720 cm^−1^, which now encompassed contributions from urethane, ester, and acrylate carbonyls, also reflected the complex multi-component structure of the prepolymer. These detailed spectral interpretations unequivocally substantiated the successful construction of the designed molecular structure.

**Figure 4:**
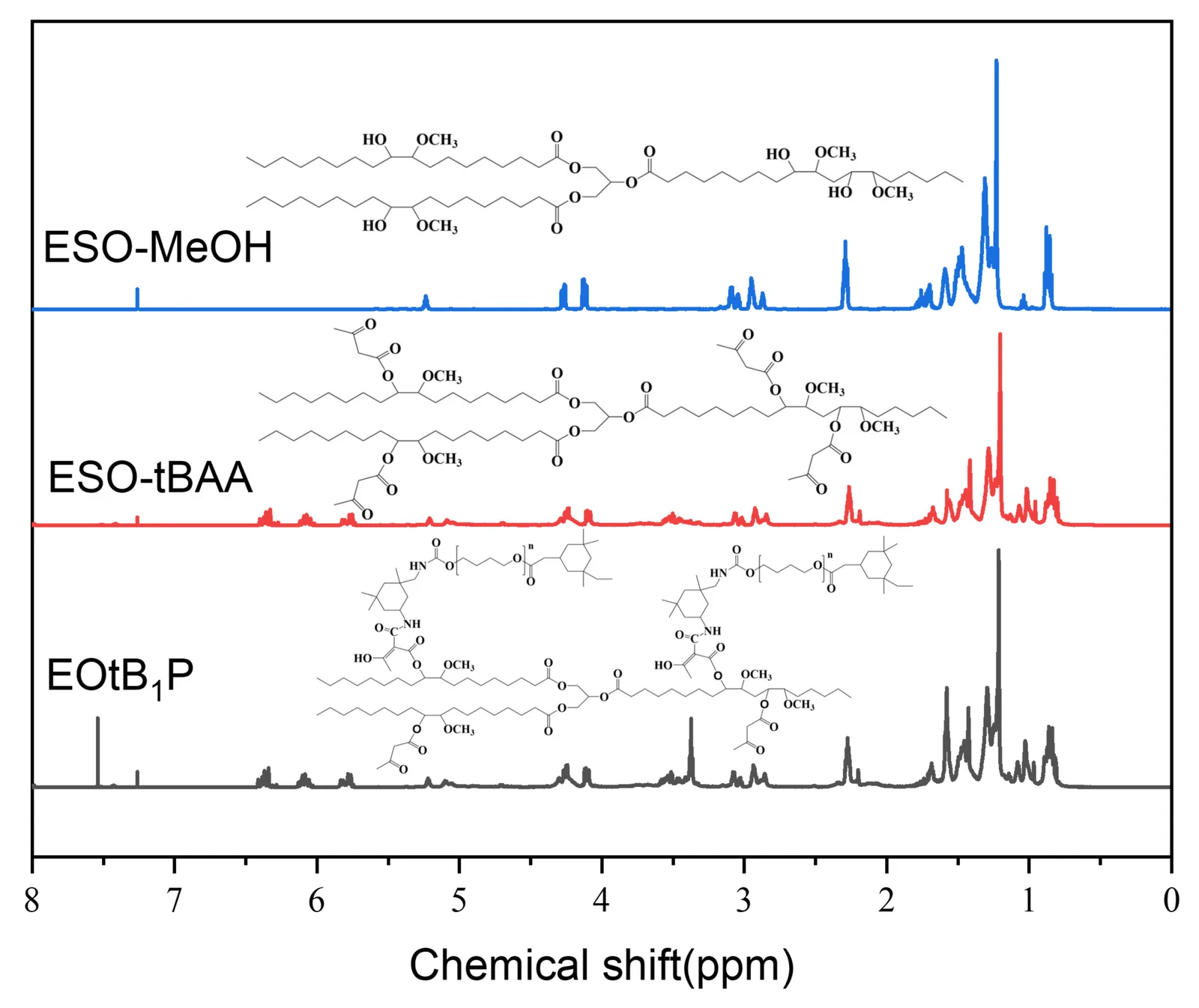
^1^H NMR spectra of ESO-MeOH, ESO-tBAA, and EOtB_x_P in CDCl_3_.

The ^1^H NMR spectra of ESO-MeOH, ESO-tBAA, and EOtB_x_P in CDCl_3_ are shown in Figure 4. In the ^1^H NMR spectrum of ESO-MeOH, the signals in the range of 2.8–3.0 ppm were attributed to the protons of the oxirane rings that had undergone ring-opening [37], while the peak at 3.3–3.4 ppm corresponded to the protons of the methoxy group (–OCH_3_) introduced by methanolysis. These observations confirmed the successful ring-opening of the epoxy groups in ESO with methanol. For ESO-tBAA, the intensity of the hydroxyl proton signal (around 2.8–3.0 ppm) decreased significantly compared with that in ESO-MeOH, indicating the consumption of hydroxyl groups via further modification. Meanwhile, new peaks appeared at approximately 2.2 ppm and 3.4 ppm, which were assigned to the methylene protons (–CO–CH_2_–CO–) adjacent to the carbonyl groups [38] and the tert-butyl protons [–C(CH_3_)_3_], respectively, of the acetoacetate moiety introduced by the transesterification reaction between ESO-MeOH and tert-butyl acetoacetate. In addition, the signal around 5.0–5.4 ppm corresponded to the methine protons (–CH–) on the glycerol backbone of the triglyceride structure. These spectral changes confirmed the successful incorporation of t-BAA into the molecular structure via nucleophilic attack of the hydroxyl groups on the carbonyl carbon of tert-butyl acetoacetate, forming new ester bonds. In the ^1^H NMR spectrum of EOtB_x_P, a new signal appeared at approximately 7.3–7.5 ppm, which was characteristic of the urethane N–H proton (–NH–CO–), confirming the formation of urethane linkages between the hydroxyl groups and isophorone diisocyanate (IPDI). The appearance of this signal, along with the disappearance of the hydroxyl proton signals, provided further evidence for the successful synthesis of the polyurethane acrylate prepolymer.

To quantitatively assess the efficiency of the ring-opening reaction, the conversion of epoxy groups was estimated by ^1^H NMR integration. The peak area corresponding to the oxirane protons (δ = 2.8–3.0 ppm) was normalized against the signal of the glycerol backbone methine protons (δ = 5.0–5.4 ppm), which remained constant throughout the reaction. Compared with pristine ESO, the relative integration of the oxirane proton signal in ESO-MeOH decreased remarkably, indicating a high epoxy conversion of approximately 96.5%. This result demonstrated that the methanolysis reaction proceeded with near-quantitative efficiency under the employed conditions (65 °C, 2 h). The subsequent transesterification with tert-butyl acetoacetate was also highly effective, as evidenced by the appearance of new peaks characteristic of the acetoacetate moiety (δ ≈ 2.2 ppm for –CO–CH_2_–CO– and δ ≈ 3.4 ppm for –C(CH_3_)_3_), confirming the successful grafting of dynamic ester precursors onto the polyol backbone.

### 3.2. Gel Content of the UV-Cured EOtB_x_P Samples

The results of the gel content for the UV cured samples are presented in Table 2.

With the n_COO_ and R value (R = n_NCO_/n_OH_) increased, the gel contents of all the samples exhibited a trend of first increasing significantly and then leveling off, reaching a maximum value (80.8%) at the highest n_COO_ and R values, indicating that the components and their stoichiometric balance were the primary conditions for achieving a complete crosslinked network structure [13,39]. As the n_COO_ and R values increased, the proportion of dynamic covalent bonds in the system increased correspondingly, which favored an increase in the gel content of the cured product. In addition, the ESO-tBAA structure was rich in reactive sites capable of participating in crosslinking, playing a key role as a multifunctional crosslinking center. An increase in its relative content significantly enhanced the average crosslinking density of the system, thereby improving the integrity of the three-dimensional network. In contrast, the EOtB_1_P sample, which had the highest PTMG content, exhibited the lowest gel content of only 66.4%. This was attributed to the “dilution effect” and the significant steric hindrance associated with the long-chain PTMG molecular chains [40]. The introduction of long-chain flexible segments, on one hand, reduced the concentration of reactive groups available for crosslinking per unit volume and, on the other hand, might inhibit the formation of effective chemical bonds between crosslinking points, thereby limiting the densification of the network structure to a certain extent. The variation in gel content directly reflected differences in the crosslinking density of the material, which in turn determined the swelling behavior and macroscopic mechanical properties of the cured product. Therefore, the rational regulation of the R value and the ratio of soft segment components were of great significance for optimizing the network structure and overall performance of the material.

### 3.3. Thermal Properties of the UV-Cured EOtB_x_P Samples

**Figure 5:**
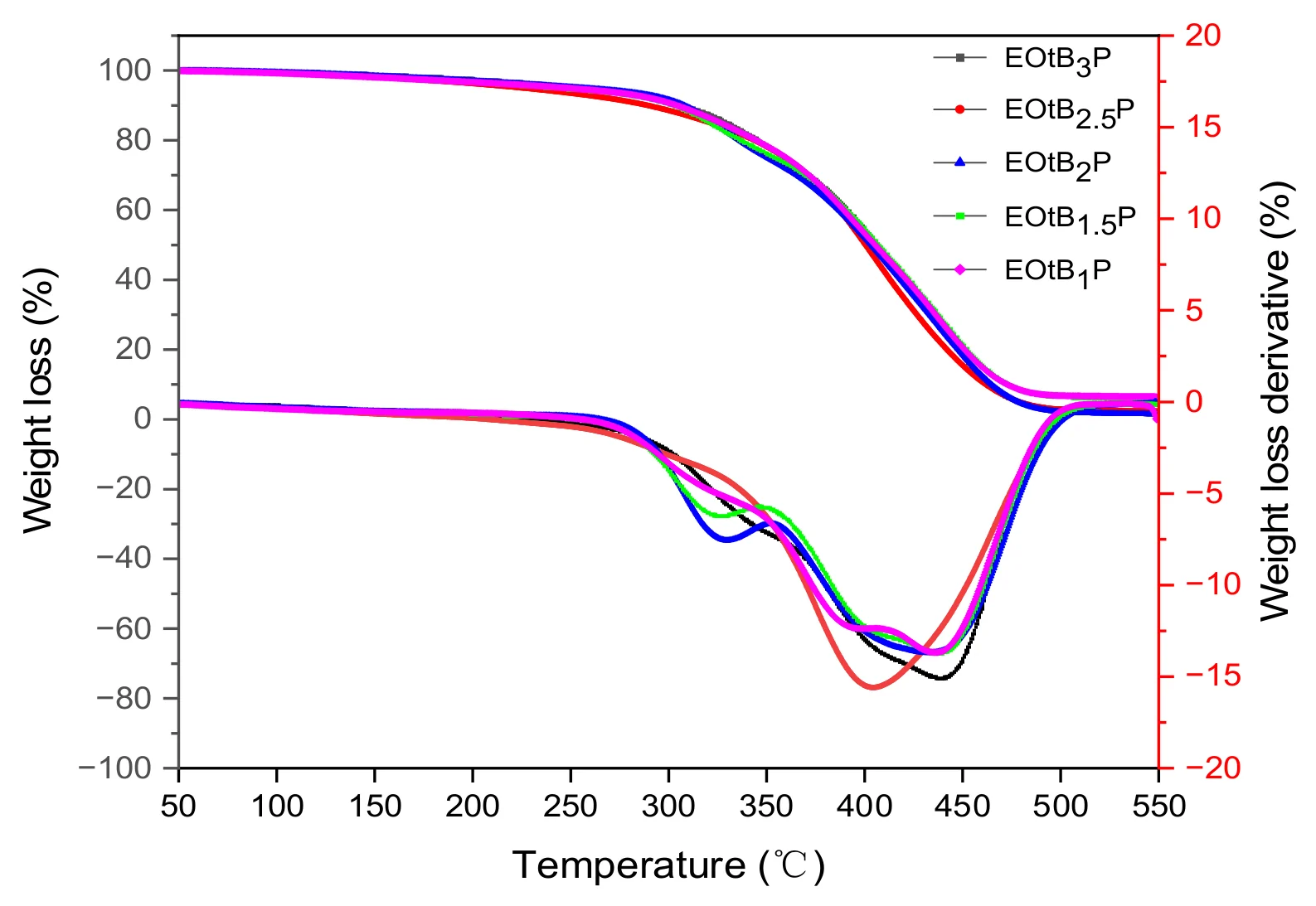
TGA and DTG curves of the UV-cured EOtB_x_P samples.

Figure 5 displays the TG and DTG curves of the UV-cured EOtB_x_P materials, with key thermal property data summarized in Table 3.

From the table, EOtB_3_P had the greatest onset decomposition temperature (T_5%_) at 254.5 °C, and EOtB_2.5_P had the smallest one at 225.5 °C, meaning that all samples remained stable below 225 °C. Meanwhile, all UV-cured samples showed high peak decomposition temperatures (T_p_), ranging from 405.0 °C to 438.9 °C, also demonstrating good overall thermal stability. The thermal stability of EOtB_x_P made it possible to be practically reprocessed, which was generally operated below 200 °C [41,42].

Further examination of the TG–DTG curves revealed that, as the proportion of dynamic ester bonds (–COO–) increased to 3 mmol and the R value to 4, both the T_5%_ and T_p_ of EOtB_3_P reached the highest values, suggesting that increasing the content of dynamic covalent bonds in the system contributed to improved thermal stability at elevated temperatures, as the increased rigid structure made the material be more heat-resistant. Where the effects of PTMG content on the thermal behaviors are concerned, however, it could be found that the samples with higher PTMG content exhibited a noticeable shoulder peak near 350 °C, which corresponded to the thermal cleavage of the polyether soft segments [43]. Simultaneously, the intensity of the high temperature main peak, attributed to the decomposition of urethane bonds and dynamic ester bonds in the hard segments, diminished, indicating that a higher proportion of soft segments diluted the thermal contribution from the hard segment network.

The observed trends in thermal stability could be directly correlated with the network structure characterized by the gel content (Table 2). Generally, samples with higher gel content, which indicated a higher crosslinking density, exhibited superior thermal stability. For example, the EOtB_3_P sample, possessing the highest gel content (80.8%), also showed the greatest onset decomposition temperature (T_5%_ = 254.5 °C) and one of the highest peak decomposition temperatures (T_p_ = 438.9 °C). This is because a more densely crosslinked network restricted segmental mobility and required more energy for the onset of thermal degradation. Conversely, the EOtB_1_P sample, with the lowest gel content (66.4%) due to the diluting effect of PTMG, displayed a lower initial decomposition temperature. This direct correlation confirmed that a well-developed, highly crosslinked network formed by the dynamic covalent bonds was crucial for enhancing the thermal resistance of these bio-based materials.

### 3.4. Mechanical Properties of the UV-Cured EOtB_x_P Samples

**Figure 6:**
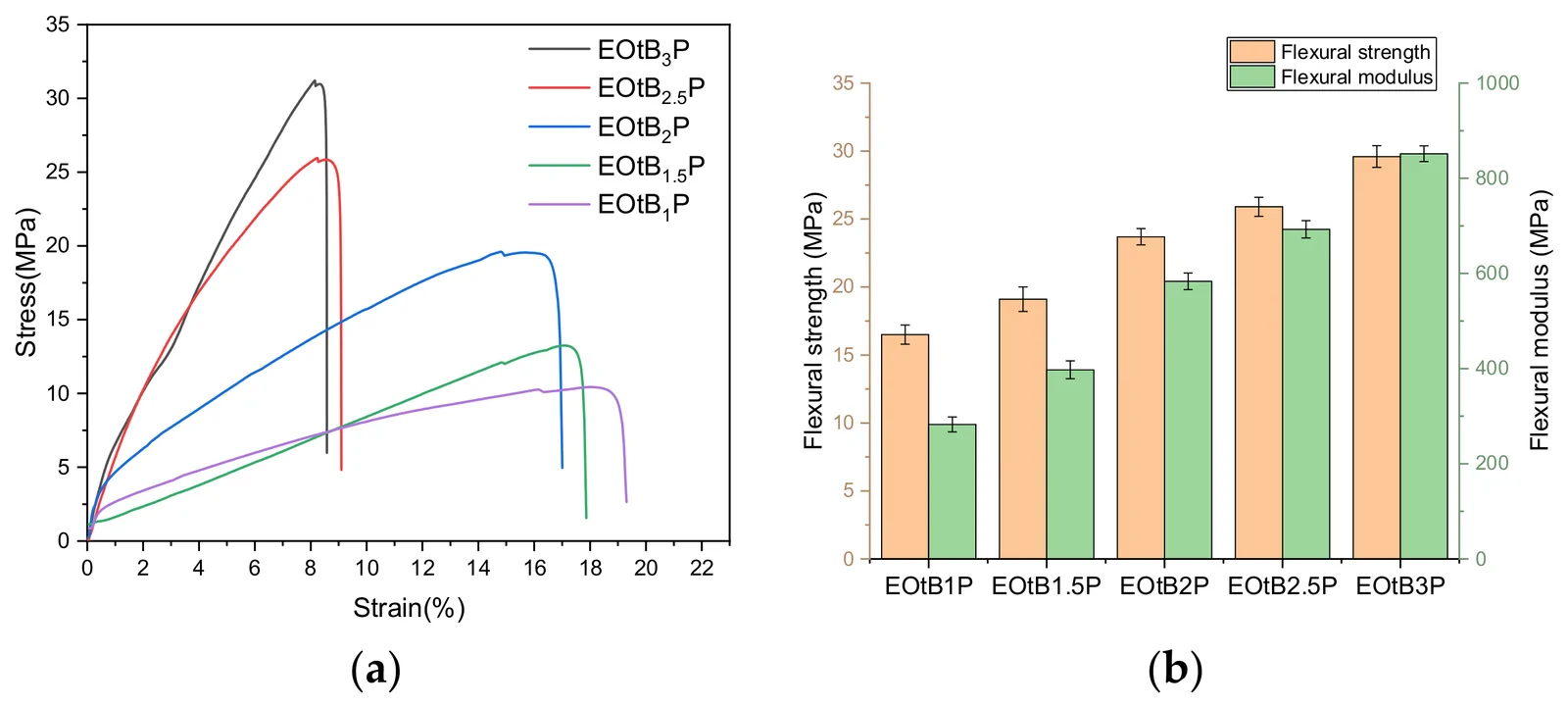
Tensile and flexural properties of the UV-cured EOtB_x_P samples: (**a**) tensile stress–strain curves, (**b**) flexural properties.

Figure 6 presents the stress–strain curves in the tensile test and the flexural properties of the UV-cured EOtB_x_P samples; the corresponding test results are summarized in Table 4.

**Table 4:** Tensile and flexural properties of the UV-cured EOtB_x_P samples.

Samples	Tensile Strength (MPa)	Tensile Modulus (MPa)	Elongation at Break (%)	Flexural Strength (MPa)	Flexural Modulus (MPa)
EOtB_1_P	10.3 ± 0.1	153.55 ± 2.7	18.25 ± 0.8	16.5 ± 0.7	282.7 ± 15.6
EOtB_1.5_P	13.1 ± 0.2	176.1 ± 0.6	17.01 ± 1.1	19.1 ± 0.9	397.5 ± 18.7
EOtB_2_P	19.4 ± 0.6	222.5 ± 1.3	16.26 ± 2.1	23.7 ± 0.6	583.4 ± 17.5
EOtB_2.5_P	25.9 ± 0.3	294.8 ± 0.2	9.26 ± 0.5	25.9 ± 0.7	692.7 ± 18.2
EOtB_3_P	31.1 ± 0.1	393.7 ± 0.4	8.57 ±1.3	29.6± 0.8	851.6 ± 16.6

As shown in Figure 6 and Table 4, with the increases in the proportion of dynamic ester bonds (–COO–) and the R value, the tensile strength and modulus of the UV-cured samples increased monotonically from 10.3 MPa and 153.55 MPa to 31.1 MPa and 393.7 MPa, respectively. This is attributed to the rigid cyclohexane structure and the exchangeable dynamic covalent bonds of EOtB_x_P. Increasing their proportion enhanced the overall rigidity and crosslinking density of the material, leading to a significant increase in tensile properties. Meanwhile, the flexural strength and modulus of the samples rose monotonically from 16.5 MPa and 282.7 MPa to 29.6 MPa and 851.6 MPa, accordingly, indicating an enhanced resistance to bending deformation and improved load-bearing capacity. Contrary to the changing trends of the strength and modulus, the elongation at break of the sample decreased gradually from 18.25% to 8.57%, indicating that the material became more brittle and exhibited poorer ductility [44]; the brittleness of the sample when fractured can also be observed in Figure 6a because no yield points appeared in the stress–strain curves.

When the R value decreased from 4 to 1.3, n _PTMG_ increased gradually from 1 mmol to 3 mmol. Theoretically, PTMG, as a long-chain flexible soft segment, could absorb and disperse stress, acting as a stress-dispersing region. Increasing PTMG content could effectively promote microphase separation; as a result, the material ductility was improved significantly. However, this simultaneously diluted the rigidity contributed by the hard segment phase, resulting in decreased strength and modulus. This is why it is difficult for the self-healing materials to have excellent self-repairing properties and high mechanical properties at the same time [4]. This study shows that adjusting the soft-to-hard segment ratio was an effective strategy for tailoring the mechanical properties of self-healable vegetable oil-based polyurethanes, enabling a transition from rigidity to toughness.

The mechanical performance of the developed EOtB_x_P resins compared favorably with other bio-based self-healing polymers reported in the literature. For instance, the tensile strength of our EOtB_3_P sample (31.1 MPa) was significantly higher than that of many previously reported castor oil-based self-healing polyurethanes, which often exhibited tensile strengths below 20 MPa [2]. Similarly, compared with other ESO-based UV-curable systems that typically showed tensile strengths in the range of 5–15 MPa [41], our materials demonstrated a marked improvement. This enhancement was attributed to the synergistic effect of the rigid IPDI-derived hard segments, the multifunctional crosslinking provided by the ESO-tBAA core, and the robust dynamic covalent network formed. While the elongation at break of our materials was relatively modest (8.57–18.25%), it was comparable to, or even higher than, some high-strength vitrimer systems [42], indicating that our strategy successfully achieved a favorable balance between rigidity and toughness, which was often challenging for crosslinked networks.

### 3.5. Self-Healing Performance of the UV-Cured EOtB_x_P Samples

**Figure 7:**
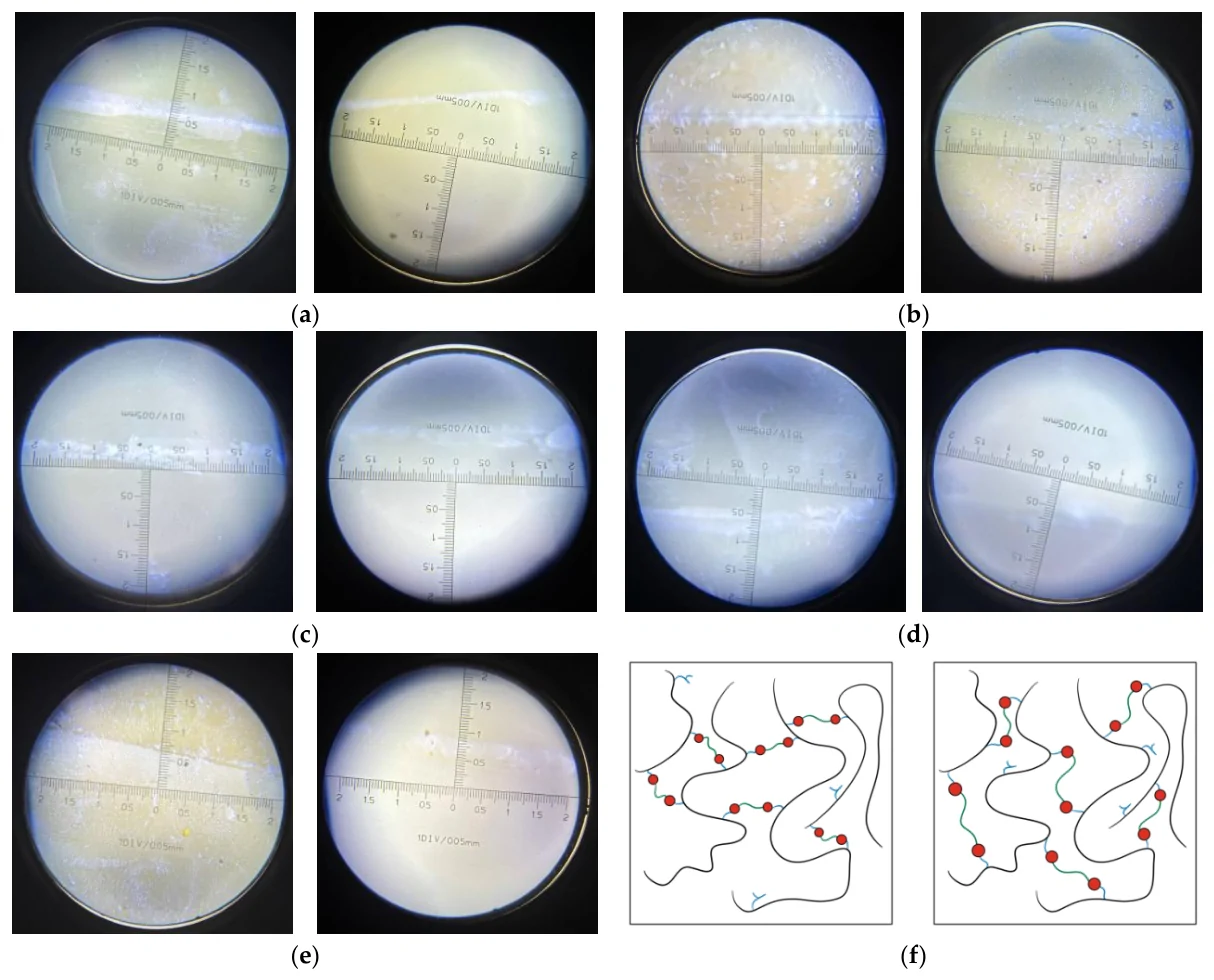
Comparison of scratches on the EOtB_x_P sample before and after repair: (**a**) EOtB_1_P; (**b**) EOtB_1.5_P; (**c**) EOtB_2_P; (**d**) EOtB_2.5_P; (**e**) EOtB_3_P; (**f**) illustration of the self-healing mechanism.

As shown in Figure 7, the self-healing performance of the UV-cured EOtB_x_P samples was verified by scratch healing tests. The scratch widths before and after healing, along with the self-healing efficiencies, are summarized in Table 5.

The results in Figure 7a–e show that all the samples exhibited significant self-healing capability. Within 30 min, the self-healing efficiency of each sample exceeded 85.4%, and the EOtB_2_P had the greatest at 94.4%. This excellent performance was mainly attributed to the traditional dynamic ester bonds [41] and the novel dynamic amide bonds in the EOtB_x_P network; the dynamic covalent bonds could endow the damaged sample with network rearrangement ability and finally undergo recombination under heating conditions. However, when the proportion of dynamic ester bonds (–COO–) was excessively high, the crosslinking density would be so great so as to restrict chain segment mobility, making it difficult for the fractured surfaces to achieve close contact and undergo sufficient exchange reactions, thereby reducing the healing efficiency. Figure 7f schematically illustrates the self-healing mechanism. The amide bonds formed by the reaction of acetoacetyl groups with isocyanate possessed lower bond energy, making them easier to cleave and exhibiting higher reactivity. Benefiting from the efficient transamidation reaction mechanism, this crosslinked network could undergo multiple network rearrangements under heating conditions without the need for an external catalyst [45]. This was exemplified by the capability to heal scratches effectively, as the dynamic bonds could break and reform multiple times to relieve stress and restore integrity, thereby endowing the material with sustained and stable self-healing capability.

### 3.6. Shape Memory Behaviors of the UV-Cured EOtB_x_P Samples

**Figure 8:**
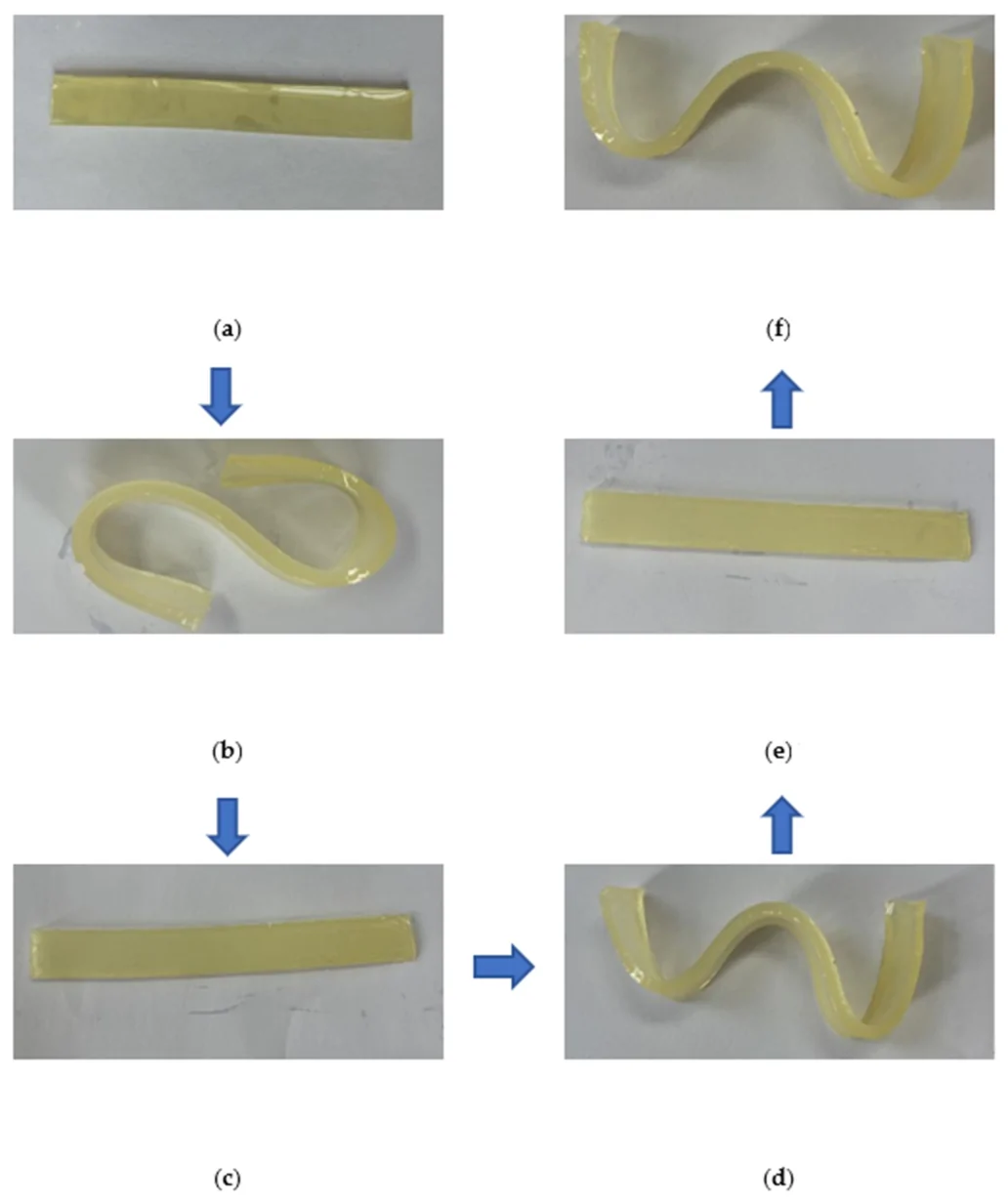
Visual demonstration of the shape memory behavior of UV-cured EOtB_2_P sample: (**a**) original rectangular shape, (**b**) temporary “S” shape, (**c**) recovery to the original rectangular shape, (**d**) ductility induced permanent “W” shape, (**e**) temporary rectangular shape, and (**f**) final recovery to the permanent “W” shape.

The molecular structure of EOtB_x_P contained abundant dynamic covalent bonds, suggesting that the UV-cured EOtB_x_P samples could be reshaped via network topological rearrangement during the reconfiguration process at elevated temperatures [42]. Based on this, the EOtB_2_P sample, which exhibited relatively overall good performance, was selected for an in-depth investigation of its shape memory behaviors. The relevant processes and results are demonstrated in Figure 8.

From the figure, it was found that the “S” shape of the sample was easy to be fixed in step 1, and could be recovered to its original rectangular shape after step 2; similarly, the “W” shape of the sample was easy to be fixed in step 3, and again it could be recovered to its original rectangular shape after step 4, and when the external force was removed in step 5, the sample could be quickly recovered to the “W” shape at 150 °C. The experimental results demonstrate that the obtained EOtB_2_P sample exhibited excellent shape memory performance. The reasons should be that the modification of ESO with methanol and t-BAA introduced reversible dynamic covalent bonds. The construction of a thermally induced phase-separated structure via IPDI and PTMG provided reversible deformability from the PTMG soft segments and a fixed phase from the IPDI hard segments. When the UV-cured sample was heat treated, the soft segment, which was much easier to move than the hard segment [46], underwent orientation and rearrangement, while the hard segments temporarily fixed the shape. Cooling locked the deformed shape. Upon reheating above the transition temperature, dynamic bond reorganization drove recovery of the original shape. Furthermore, PTMG exhibited good flexibility, and after its crystalline melting, it provided a fast and efficient driving force for shape recovery, resulting in a very high shape recovery ratio for the material.

### 3.7. Wettability of the UV-Cured EOtB_x_P Samples

**Figure 9:**
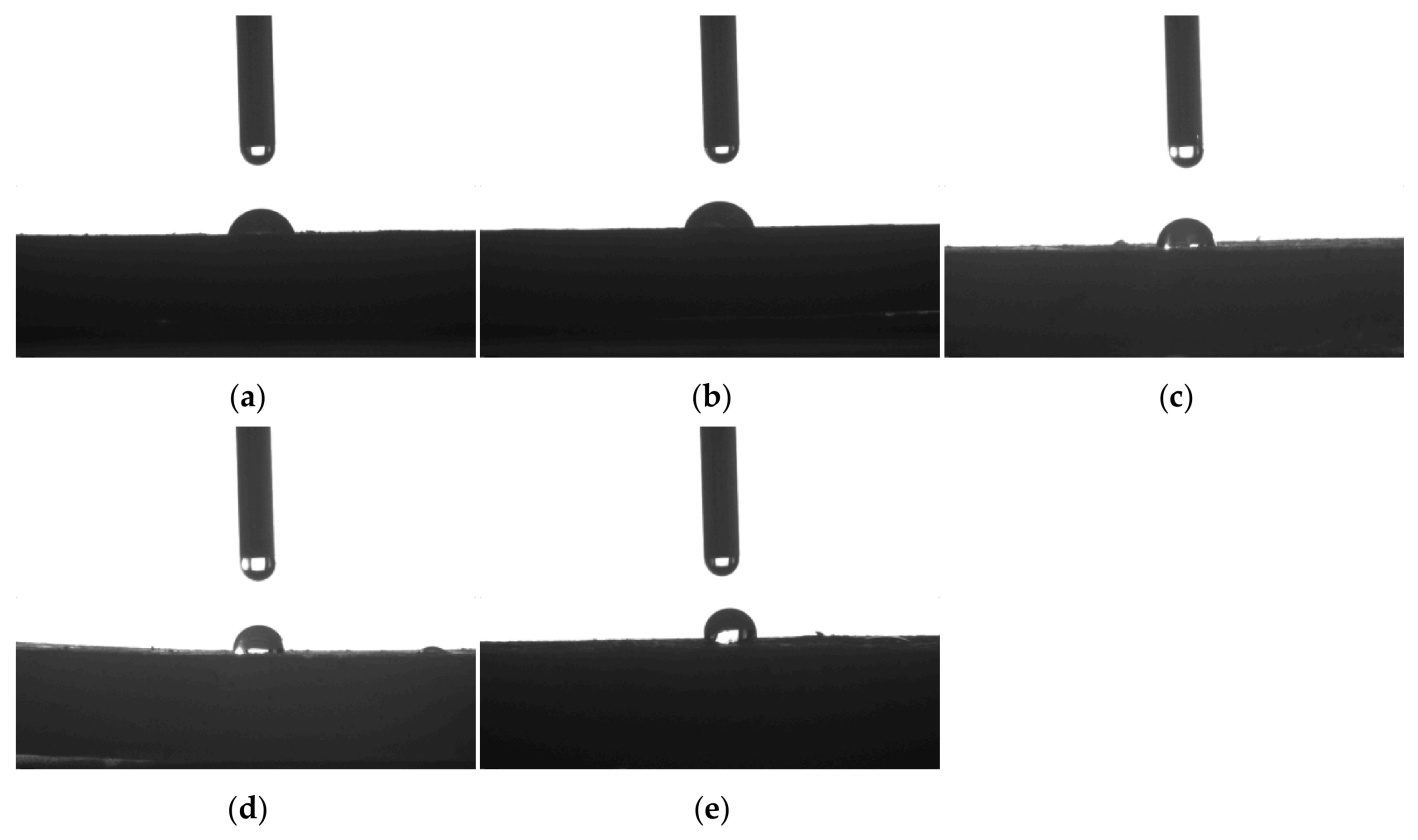
Contact angles of the UV-cured EOtB_x_P samples: (**a**) EOtB_1_P; (**b**) EOtB_1.5_P; (**c**) EOtB_2_P; (**d**) EOtB_2.5_P; (**e**) EOtB_3_P.

Figure 9 shows the water contact angle images on the surfaces of the obtained samples, with the corresponding data listed in Table 6.

It was observed that the water contact angles of the EOtB_2.5_P and EOtB_3_P samples were greater than 90°, showing hydrophobic characteristics, while the contact angles of the other samples were all less than 90°, indicating hydrophilic characteristics. In EOtB_x_P, the long-chain aliphatic hydrocarbons and cyclic structures were hydrophobic, whereas the introduced dynamic covalent bonds and hydroxyl groups were relatively hydrophilic. With increasing R value, the hydrophobic aliphatic chains contributed by the R value became dominant, leading to an overall enhancement in the hydrophobicity of the material and an increase in water contact angle. As the PTMG content increased, the contact angle decreased from 95.2° to 73.4°. This was attributed to the fact that the ether bond (–C–O–C–) of PTMG was a hydrophilic group capable of forming hydrogen bonds with water molecules. Meanwhile, an increased content of PTMG meant a reduced content of hard segments, which contributed to the enhancement of the hydrophobicity of the produced photocured sample [22]. Increasing the PTMG content was thus the most definitive and effective means of reducing the surface hydrophobicity and decreasing the water contact angle of the material. More hydrophilic ether bonds enriched the surface, increasing the surface energy of the material and making it easier for water droplets to spread.

## 4. Conclusions

In this study, a series of bio-based UV-curable polyurethane acrylate resins (EOtBXP) were successfully synthesized from epoxidized soybean oil. The systematic investigation of their structure–property relationships led to the following conclusions.

Composition–Structure–Performance Relationship: A clear quantitative correlation was established. Increasing the dynamic ester bond content (from 1 to 3 mmol) and the R value (from 1.3 to 4) systematically increased the crosslinking density (evidenced by gel content rising from 66.4% to 80.8%). This led to a proportional increase in mechanical properties (tensile strength from 10.3 to 31.1 MPa, flexural strength from 16.5 to 29.6 MPa) and thermal stability (onset decomposition temperature up to 254.5 °C). However, this came at the cost of reduced ductility (elongation at break decreased from 18.25% to 8.57%).

Self-Healing Efficiency: The introduction of dynamic ester and amide bonds endowed the materials with excellent self-healing capabilities. A maximum scratch healing efficiency of 94.4% was achieved for the EOtB2P sample, demonstrating that a balanced crosslinking density was crucial for optimal network rearrangement.

Functionality: The materials also exhibited excellent shape memory behavior and tunable surface wettability (water contact angle ranging from 73.4° to 95.2°), which were directly governed by the soft/hard segment ratio.

In conclusion, this work successfully demonstrated a novel and effective strategy for preparing high-performance, multifunctional bio-based polymers, providing a solid foundation for the development of sustainable smart materials.

## Figures and Tables

**Table 1:** Formulation of the EOtB_x_P.

	COO _ESO-tBAA_	NCO _IPDI_	OH(PTMG)	R
Samples	(mmol)	(mmol)	(mmol)	R = n_NCO_/n_OH_
EOtB_1_P	1	4	3	1.3
EOtB_1.5_P	1.5	4	2.5	1.6
EOtB_2_P	2	4	2	2
EOtB_2.5_P	2.5	4	1.5	2.7
EOtB_3_P	3	4	1	4

**Table 2:** Gel contents of the UV-cured EOtB_x_P samples.

Samples	EOtB_1_P	EOtB_1.5_P	EOtB_2_P	EOtB_2.5_P	EOtB_3_P
*C*_gel_ (%)	66.4 ± 0.1	79.8 ± 0.5	78.4 ± 0.4	79.4 ± 0.1	80.8 ± 0.2

**Table 3:** Main thermal parameters of the UV-cured EOtB_x_P samples.

Samples	T_5%_ (°C)	*T*_p_ (°C)	*w*_char_ (%)
EOtB_1_P	247.9	436.1	6.46
EOtB_1.5_P	241.5	438.5	5.96
EOtB_2_P	253.5	432.5	1.48
EOtB_2.5_P	225.5	405.0	2.32
EOtB_3_P	254.5	438.9	2.67

**Table 5:** Self-healing properties of the UV-cured EOtB_x_P samples.

Samples	Scratch Width D_1_ Before Healing (μm)	Scratch Width D_2_ After Healing (μm)	Self-Healing Efficiency R_r_ (%)
EOtB_1_P	24.6	3.6	85.4
EOtB_1.5_P	22.4	1.8	92.0
EOtB_2_P	19.2	1.1	94.4
EOtB_2.5_P	21.7	2.7	87.6
EOtB_3_P	18.4	2.3	87.5

**Table 6:** Water contact angles of the UV-cured EOtB_x_P samples.

Samples	EOtB_1_P	EOtB_1.5_P	EOtB_2_P	EOtB_2.5_P	EOtB_3_P
angles/°	73.4 ± 0.5	76.6 ± 0.7	87.3 ± 0.2	90.5 ± 0.3	95.2 ± 0.4

## Data Availability

The original contributions presented in this study are included in the article. Further inquiries can be directed to the corresponding authors.
